# Bone-Sparing Hip Arthroplasty in Young and Active Patients: A Narrative Review

**DOI:** 10.3390/jcm15155894

**Published:** 2026-07-28

**Authors:** Roberto Perulli, Calogero Graci, Raffaele Vitiello, Alessandro El Motassime, Roberta Righini, Alessandro Bumbaca, Carlotta Genesini, Marco Libertini, Giulio Maccauro, Riccardo Giorgino

**Affiliations:** 1 Department of Orthopedics and Geriatrics Sciences, Università Cattolica del Sacro Cuore, 00168 Rome, Italy; rob.perulli@gmail.com (R.P.); calogerograci@hotmail.it (C.G.); raffaele.vitiello@policlinicogemelli.it (R.V.); alessandroelmotassime@gmail.com (A.E.M.); riro.righini@gmail.com (R.R.); alessandro.bumbaca@gmail.com (A.B.); carlotta.genesini15@gmail.com (C.G.); marco.libertini@esercito.difesa.it (M.L.); 2 Department of Orthopedics, Ageing and Rheumatological Sciences, Fondazione Policlinico Universitario Agostino Gemelli IRCCS, 00168 Rome, Italy; 3 Department of Experimental Medicine and Surgery, Defence Institute for Biomedical Sciences, Via Santo Stefano Rotondo 4, 70124 Rome, Italy; 4 Department of Precision and Regenerative Medicine and Ionian Area (DiMePreJ), University of Bari Aldo Moro, 20157 Bari, Italy; dr.riccardogiorgino@gmail.com; 5 IRCCS Ospedale Galeazzi Sant’Ambrogio, 00184 Milan, Italy

**Keywords:** hip resurfacing arthroplasty, short-stem total hip arthroplasty, bone preservation, stress shielding, periprosthetic bone remodeling, revision hip arthroplasty, isoelastic acetabular cup, young adult, cementless fixation, metal-on-metal, ceramic-on-ceramic, dual mobility, young and active patient

## Abstract

Bone stock preservation in young and active patients undergoing hip arthroplasty is the principal determinant of reconstructive complexity at revision, an event that national registry data identify as more likely for most young individuals, with a lifetime revision risk up to 35%. Standard diaphyseal stems, despite their proven longevity in elderly cohorts, produce predictable proximal stress shielding that depletes the metaphyseal envelope over time, narrowing the surgical options available at every subsequent intervention. Two femoral strategies have evolved in response: hip resurfacing arthroplasty and short-stem total hip arthroplasty. Each addresses proximal bone loss through distinct biomechanical principles and with different patient selection criteria, complication profiles and revision implications. Although acetabular bone preservation has been comparatively less studied in the literature, it remains clinically relevant because it may affect bone stock quality at the time of revision. We synthesize the available evidence on both femoral and acetabular bone-sparing strategies, with respect to patient selection, surgical technique, survivorship, patient-reported outcomes, complications and revision implications. Our goal is to provide a structured clinical reference for implant selection in this demanding patient population. A comprehensive literature review was performed using PubMed and Google Scholar (October 2000–May 2026) and 99 peer-reviewed studies were included and synthesized using a structured narrative approach.

## 1. Introduction

Femoral bone preservation at the index procedure could influence the range of surgical options available at revision. This consideration is particularly relevant in younger patients, in whom registry-based data indicate a substantially increased lifetime risk of revision, with revision rates up to 35% in selected cohorts [1]. Standard diaphyseal-fixation stems, despite their proven longevity in elderly cohorts, transfer load preferentially to the femoral diaphysis, producing a predictable pattern of proximal stress shielding that depletes the metaphyseal envelope over time. The clinical consequences of this biological debt show at revision: calcar resorption, cortical thinning and a compromised substrate for implant anchorage [2].

Two distinct surgical philosophies have evolved in response: hip resurfacing and short stems. Hip resurfacing arthroplasty (HRA) preserves the femoral head and neck in their entirety by capping the native femoral head with a shell prosthesis while avoiding femoral canal preparation. Most of the available evidence relates to metal-on-metal HRA designs, whereas alternative materials such as ceramic resurfacing remain more limited and are still emerging. In the cited high-resolution DXA region-free analysis study, HRA was the only arthroplasty configuration evaluated that showed no measurable periprosthetic bone loss at two years, with a concurrent BMD increase of approximately 34% across 30% of the proximal metaphysis, including the cortical bone of the medial femoral neck [3]. Short-stem total hip arthroplasty (THA), defined by implant lengths below 120 mm, achieves metaphyseal fixation while sparing the diaphysis entirely; its defining biomechanical characteristic is proximal load transfer through fit-and-fill anchoring, which has been shown to reduce calcar atrophy at 13 years to 5.16% in Gruen zone 7 compared with 33.04% for traditional cobalt–chrome diaphyseal designs [2]. Both strategies target proximal femoral preservation yet differ in execution, technical demands and the degree of bone conservation ultimately achieved. Although preservation of proximal femoral bone stock is generally considered advantageous for future reconstruction, direct evidence demonstrating that improved periprosthetic bone preservation consistently translates into less complex revision surgery remains limited, with most supporting data derived from densitometric rather than clinical revision studies. Comparative data on long-term densitometric outcomes, procedure-specific complication profiles and implications at revision remains limited and methodologically heterogeneous.

A Bayesian network meta-analysis synthesizing 72 studies across 793,593 patients found no significant differences in overall complication rates between hip resurfacing arthroplasty (HRA), short-stem total hip arthroplasty and conventional THA. However, when conventional THA was used as the reference comparator, HRA was associated with a significantly lower risk of periprosthetic joint infection (RR 2.14, 95% CI 1.33–5.44) and dislocation (RR 13.45, 95% CI 3.37–98.21). Both bone-preserving femoral techniques therefore emerge as rational alternatives to conventional stems especially in the young active patient. These procedures also carry specific complications. Short stems may compromise fixation in Dorr C femurs, predisposing to periprosthetic fracture due to inadequate metaphyseal engagement. Hip resurfacing, relying on metal-on-metal bearings, raises concerns regarding metallosis, adverse local tissue reactions and femoral neck fracture. Furthermore revision rates are disproportionately elevated in female and elderly patients, confirming that patient selection remains the dominant outcome modifier [4].

The acetabular side of the equation has received comparatively less attention in the bone-preservation literature, although preservation of periacetabular bone stock remains clinically relevant at the time of revision surgery. From a biomechanical perspective, isoelastic monoblock press-fit cups, designed with an elastic modulus closer to that of cancellous bone, have been associated with reduced stress-shielding mediated bone loss and more favorable periacetabular bone remodeling compared with modular titanium shells across DeLee and Charnley zones, as documented in clinical and experimental studies. In parallel, wear-related osteolysis represents a distinct biological pathway of bone loss. This is addressed by modern tribological improvements, including vitamin E-infused highly cross-linked polyethylene (VEHXLPE) liners and ceramic-on-ceramic bearings, which aim to reduce wear debris osteolysis rather than mechanically mediated bone loss. These two mechanisms, stress shielding mediated bone loss and debris driven osteolysis, therefore represent complementary but distinct targets in acetabular bone preservation strategies [5].

The present narrative review summarizes the available evidence on femoral and acetabular bone-sparing arthroplasty addressing: patient selection, surgical technique, implant survivorship, patient-reported outcomes, complications and revision implications for each approach. The aim is to review the available evidence on bone-sparing strategies and support implant selection decisions in young and active patients undergoing primary hip arthroplasty. Although bone preservation represents the conceptual foundation of these procedures, the clinical relevance of bone stock preservation should be interpreted within a broader concept that becomes increasingly relevant in young arthroplasty patients: the preservation of future reconstructive options. In this context, the success of a bone-preserving procedure should not be judged solely by the quantity of retained bone, but by its ability to facilitate future revision strategies while maintaining functional outcomes.

## 2. Methods

A comprehensive literature search was conducted across PubMed and Google Scholar for studies published between October 2000 and May 2026. Search strategies were developed using a combination of MeSH terms and free-text keywords. For PubMed, the following search string was used: (“hip resurfacing arthroplasty” OR “short-stem total hip arthroplasty” OR “bone preservation” OR “stress shielding” OR “periprosthetic bone remodeling” OR “revision hip arthroplasty” OR “isoelastic acetabular cup” OR “young adult” OR “cementless fixation” OR “metal-on-metal” OR “dual mobility” OR “cost-effectiveness” OR “health economics” OR “economic evaluation” OR “cost analysis” OR “cost-utility”). Boolean operators (AND, OR) were used where appropriate to refine the search strategy and increase specificity. Equivalent keyword-based searches were applied in Google Scholar. Additional relevant studies, including health-economic analyses, were identified through targeted reference screening where appropriate. Eligibility criteria included peer-reviewed journal articles reporting on hip resurfacing arthroplasty, short-stem total hip arthroplasty, or acetabular bone preservation strategies in adult patients. Priority was given to registry-based studies, systematic reviews, meta-analyses and randomized controlled trials. Only articles published in English were included. Case reports, conference abstracts, cadaveric studies and non-peer-reviewed publications were excluded. Study selection involved screening of titles, abstracts and full texts for relevance. The selection process was performed by a single reviewer, based on predefined eligibility criteria and relevance to the review topics. No formal risk-of-bias or quality appraisal tool was applied. This is a narrative review with a structured, procedure-based synthesis. This review has several limitations. First, study selection and data extraction were performed by a single reviewer without independent screening or assessment of inter-rater agreement. Although predefined eligibility criteria were applied consistently throughout the review process, this methodological approach may have introduced selection bias and reduced reproducibility. Second, as a narrative review, no formal risk-of-bias assessment or quantitative synthesis was performed. Therefore, the conclusions should be interpreted as a structured synthesis of the available evidence rather than as pooled estimates of comparative effectiveness. The literature references of identified papers were also searched in order to find further relevant articles. A total of 99 peer-reviewed articles were included in the final analysis. This narrative review follows a procedure-based structure, HRA and short-stem THA are addressed independently as femoral bone preservation strategies. Each technique has its dedicated subsections covering: bone preserving rationale, patient selection, surgical technique, implant survivorship, patient-reported outcomes, procedure-specific complications and revision implications. This scheme has been similarly applied to acetabular bone preservation.

## 3. Femoral Bone-Sparing

### 3.1. Hip Resurfacing Arthroplasty

#### 3.1.1. Bone Preserving Rationale

The biomechanical case for HRA rests on a validated principle: by retaining the native femoral head and confining fixation to the head–neck metaphyseal junction, the procedure eliminates diaphyseal load transfer entirely. The consequence, measured by DXA region-free analysis, is not merely the absence of stress-shielding mediated bone loss but an active densitometric gain. More specifically, HRA was associated with an approximately 34% increase in BMD across 30% of the proximal femoral metaphysis at two years, an effect seen with no other arthroplasty configuration [2]. Additionally, by preserving both femoral head and neck, HRA reconstitutes the anatomical geometry responsible for soft-tissue tension, which translates into dislocation rates that are an order of magnitude lower than those recorded for all stemmed designs [4]. Although HRA necessarily employs larger acetabular components to accommodate the femoral head dimensions, periacetabular bone stock does not appear to be disproportionately compromised. A prospective randomized controlled trial comparing an HRA press-fit cup with a standard threaded titanium acetabular component indicated significantly better BMD preservation in favor of HRA at 24 months in three of five periacetabular regions of interest, suggesting that the bone-preserving properties of resurfacing may extend to the acetabular side [6]. These biomechanical advantages should nevertheless be interpreted within the context of contemporary clinical practice, where HRA is generally reserved for a relatively narrow group of carefully selected patients because long-term outcomes remain highly dependent on patient selection and surgical expertise.

#### 3.1.2. Patient Selection

Appropriate patient selection remains the single best determinant of long-term success in HRA, essential criteria are outlined in Table 1. Registry data consistently identify male sex, osteoarthritis as the primary diagnosis and a femoral head size exceeding 48 mm as the triad most reliably associated with favorable implant survival. Among patients meeting these criteria, the largest multicenter series (n = 11,382, age ≤ 50 years, 27 high-volume centers) reports 10-year implant survivorship of 95% rising to 99% in men, with 22-year survivorship of 90% when poorly performing designs are excluded [7]. Also in the Australian Orthopedic Association National Joint Replacement Registry (AOANJRR) males with osteoarthritis undergoing HRA with a head component ≥ 50 mm had a ten-year cumulative revision rate of approximately 5%, whereas hips with a femoral component ≤ 44 mm carried a more than fivefold greater risk of revision compared to those ≥ 55 mm, independent of sex [8]. The UK National Joint Registry similarly reported cumulative survivorship of 92.05% (95% CI 91.63–92.44%) at ten years and 88.98% (95% CI 89.32–89.6%) at fifteen years across all comers, a figure driven upward when selection is restricted to low-risk males. Current evidence supports the use of a 48 mm threshold as a practical lower limit for patient selection. [9].

Female sex constitutes a relative contraindication, primarily through anatomical rather than biological mechanisms. Women present more frequently with smaller head sizes, higher rates of developmental dysplasia of the hip (DDH) and reduced bone mineral density, all of which predispose to edge-loading of the metal-on-metal bearing and, consequently, adverse reactions to metal debris (ARMD). A systematic review by Haughom et al. documented significantly higher rates of ARMD, aseptic loosening and revision in women undergoing metal-on-metal resurfacing [10]. The Canadian Arthroplasty Society analysis of 2773 HRAs confirmed female sex as an independent risk factor for early failure. Contemporary practice therefore restricts HRA to male patients with a projected head size ≥ 48 mm, with most high-volume centers also excluding women of childbearing age due to the unresolved question of transplacental metal ion transfer [11].

Menopausal status should be considered when evaluating female candidates for HRA. Estrogen deficiency after menopause accelerates bone turnover and reduces proximal femoral bone mineral density, potentially compromising femoral neck strength. Although menopause is not an independent contraindication to resurfacing arthroplasty, the associated decline in bone quality may increase the risk of femoral neck fracture and compromise long-term implant fixation. This may be particularly relevant in women undergoing HRA at a younger age who are expected to retain their implant beyond the menopausal transition. Therefore, patient selection should consider not only chronological age but also menopausal status, bone quality, DEXA findings when indicated and other risk factors for osteoporosis [12].

Age at the time of surgery should be considered from a functional standpoint. Registry data from the AOANJRR show cumulative survivorship of 90.3% and 91.4% at fifteen years for males with osteoarthritis aged under 55 and 55–65 years, respectively, supporting extension of the indication to patients up to 65 years in experienced hands. Two patients over 65 carry a disproportionately high early revision risk (3.1% at one year vs. 1.2% in the under-55 group), primarily driven by perioperative fracture in the setting of reduced bone quality [8].

Absolute contraindications include renal insufficiency, given impaired metal ion clearance, systemic metal hypersensitivity, severe osteoporosis (T-score below −2.5), avascular necrosis involving more than one third of the femoral head and any condition precluding reliable cement–bone integration at the femoral interface. Femoral cysts, Legg–Calvé–Perthes disease and slipped capital femoral epiphysis are additional relative contraindications given their propensity to distort proximal femoral anatomy and compromise implant positioning [13].

#### 3.1.3. Preoperative Planning and Surgical Technique

Meticulous preoperative templating is essential for HRA. The surgeon must calculate the native neck-shaft angle to define the target angle, optimally within 135–145°, and plan femoral component inclination and anteversion. Finite element analysis across five orientations indicated a minimized contact pressure with 15° valgus orientation (88.94 MPa) which also promoted a more physiological stress distribution [14]. Femoral component size should match native head dimensions (at least 48 mm). Acetabular component size should be 6 mm higher than femoral size, while its orientation should target 40 ± 5° of inclination and 20 ± 5° of anteversion. Combined version must be assessed explicitly, as insufficient cup anteversion below 5° carries an odds ratio of 6.5 (95% CI 3.0–14.3) for unsafe serum metal ion elevation. Femoral head cysts exceeding 1 cm in diameter must be identified preoperatively, as they compromise fixation quality and can constitute an absolute contraindication to resurfacing [8,9,15].

No surgical approach has demonstrated superiority in functional outcome. The posterior approach offers the most extensive exposure and greatest surgeon familiarity, but carries elevated osteonecrosis risk through retinacular vessel compromise and predisposes to varus-anteverted femoral positioning. The anterior (Hueter) approach preserves vascularity and enables reliable supine cup positioning, at the cost of a learning curve of approximately 50 procedures and specialized instrumentation. The lateral (Hardinge) approach reduces vascular risk but produces Trendelenburg gait in up to 20% of patients. The trochanteric flip osteotomy provides optimal exposure with an intact posterior capsule, yet incurs non-union rates of 7–8% and restricts early weight-bearing. As always approach selection should be governed by surgeon experience and individual patient morphology [16].

Femoral component fixation remains debated. Cement penetration exceeding 30% of femoral head volume reduces local perfusion, with osteonecrosis identified histologically in up to 88% of retrieved specimens [17]. Fully porous-coated uncemented fixation eliminates late non-traumatic loosening and reduces early fracture rates, though it demands precise technique and eventually an active perioperative bone density management program [18].

Computer-assisted navigation may improve reliability by reducing stem-shaft angle outliers from 48% to 33% and radiographic femoral loosening at ten years from 12.7% to 2.3% [19]. The value of navigation decreases in experienced high-volume surgeons; its benefit is most pronounced during the learning curve, where it can accelerate the process and in patients with distorted proximal femoral anatomy. The learning curve for HRA is considerable: reliable component positioning requires upwards of 100 cases and centers performing fewer than 100 procedures annually report significantly higher failure rates [9].

#### 3.1.4. Implant Survivorship

Survivorship data for contemporary HRA, restricted to well-performing designs in appropriately selected populations, are consistently positive. The international high-volume centers dataset (n = 11,382, patients ≤ 50 years) reports overall survivorship of 95% at 10 years and 90% at 22 years [7]. A dedicated minimum 15-year follow-up study of 472 consecutive HRA cases in men confirmed survivorship of 95.1% overall, with femoral head sizes ≥ 48 mm achieving 95.8% compared with 91.3% for those below the threshold, a difference of 4.5 percentage points attributable solely to component size [20]. McMinn et al., in an analysis of 3095 Birmingham Hip Resurfacings, reported survivorship of 99%, 97% and 96% at 5, 10 and 13 years, respectively, with the best outcomes concentrated in men under 55 years with primary osteoarthritis [21]. Conversely, modern THA has shown reliable long-term survivorship in a wider range of patients. Bayliss et al. documented implant survival rates of 95.6% at 10 years and 85.0% at 20 years after THA. Therefore, although HRA can provide excellent durability in carefully selected patients treated in experienced centers, conventional THA still offers more consistent survivorship and a lower revision risk overall. For this reason, patient selection remains a key factor when considering resurfacing arthroplasty [1].

#### 3.1.5. Patient-Reported Outcomes

Functional outcomes following HRA, assessed through validated patient-reported outcome measures, are broadly equivalent to those recorded after THA in the comparative literature. Smith et al. observed, in a meta-analysis and systematic review, that PROMs after HRA are equivalent to or marginally superior to those after THA, although HRA was associated with higher rates of heterotopic ossification and revision surgery in the pooled analysis [22]. A more recent systematic review and meta-analysis by Za et al. confirmed comparable or slightly superior functional results for HRA in selected patients, particularly regarding return to high-level physical activity and overall patient satisfaction [23]. In a single-surgeon Canadian cohort with a mean follow-up of 12.4 ± 1.4 years, median Harris Hip Score (HHS) was 93.9 in males and 93.6 in females (*p* = 0.27), while median UCLA Activity Score was 8.2 in males and 7.2 in females (*p* < 0.001); median satisfaction on a 100 mm visual analogue scale (VAS) was 81.9 and 81.3, respectively (*p* = 0.35) [11]. These figures align closely with prior independent ten-year series: Treacy et al. reported a median UCLA Activity Score of 7.0 (IQR 5.0–8.0) in 144 BHRs at minimum ten-year follow-up and Holland et al. similarly documented a median UCLA of 7 at the ten-year review in a non-designer single-surgeon series [24,25]. In patients aged 35 years or younger, a cohort where the functional stakes are the highest, HRA conferred UCLA activity scores of 8.4 ± 1.6 postoperatively, modified Harris Hip Scores of 94.4 ± 8.2 and HOOS-JR of 96.5 ± 6.7, with zero revisions at five years in 76 hips followed by a single high-volume arthroplasty surgeon [13]. Return-to-sport rates reach 90.9% (95% CI 82.2–96.9) at four years follow-up in a 2023 meta-analysis by Magan et al. [26]. Remarkably in a cohort of 48 Ironman athletes undergoing HRA at a mean age of 44.8 years, return-to-sport was shown in 94% of patients who continued to perform high impact activities after surgery [27]. These findings collectively support the view that HRA reliably restores a level of function difficult to reach with conventional total hip arthroplasty (THA), where higher-impact activity is more frequently discouraged. Although data must be interpreted with the awareness that they are influenced not only by patient selection but also by implant design, patient expectations, preoperative activity level and center experience, factors that are systematically more abundant in HRA cohorts.

#### 3.1.6. HRA-Specific Complications

Adverse local tissue reactions (ALTRs) and metallosis constitute the most consequential class-specific complication of metal-on-metal hip resurfacing arthroplasty (MoM HRA). MoM bearing surfaces release cobalt and chromium ions and nanometric wear particles into the periprosthetic environment and systemic circulation. Macrophage-mediated inflammatory responses can lead to chronic synovitis, soft-tissue necrosis, osteolysis and implant loosening, while lymphocyte-dominated immune reactions underpin the histological pattern of aseptic lymphocyte-dominated vasculitis-associated lesions (ALVAL). Mabilleau et al. identified these reactions as a major concern in MoM resurfacing due to the risk of pseudotumor formation and extensive soft-tissue damage [28].

Grammatopoulos et al. demonstrated that among failed metal-on-metal hip resurfacings with confirmed ARMD, elevated whole-blood cobalt and chromium concentrations were observed in approximately 82% and 53% of patients, respectively. However, histologically significant ALVAL reactions were also identified in a substantial proportion of patients despite metal ion concentrations within conventionally accepted ranges, highlighting the limited sensitivity of serum metal ion measurements when used in isolation for detecting adverse local tissue reactions [29].

In clinical practice, whole blood cobalt and chromium concentrations are interpreted in a risk-stratified manner, with commonly used reference ranges of <2 μg/L indicating low concern, 2–7 μg/L intermediate concern, and >7 μg/L high concern. However, these thresholds show limited diagnostic accuracy when used in isolation, particularly in the presence of soft-tissue reactions or well-positioned but biologically reactive implants.

Accordingly, surveillance should be multimodal, integrating clinical assessment, serial radiographs and trends in serum metal ion levels rather than single measurements. MARS-MRI is recommended in patients with persistent or unexplained symptoms, rising or persistently elevated metal ion levels, or suspected soft-tissue abnormality on clinical or radiographic evaluation and is particularly indicated when there is discordance between clinical findings and ion measurements, as emphasized in critical reviews of MoM surveillance strategies [30]. The increasing recognition of ARMD as a major failure mode has substantially reduced indications for MoM HRA and contributed to a global decline in MoM implant utilization.

Femoral neck fracture represents another feared early complication, with an incidence ranging from 0.5% to 2% across published series, most commonly occurring within the first six postoperative months. Superior notching of the femoral neck, varus component positioning, subcapital cyst formation and female sex with small component diameters are established risk factors; notching alone has been shown to significantly reduce femoral neck vascularity. The surgeon learning curve exerts an independent and modifiable influence on early fracture risk, with early-career cases demonstrating disproportionately higher rates relative to subsequent experience [31].

#### 3.1.7. Revision Rate and Revision Surgery

Palazzuolo et al. in their meta-analysis of HRA versus THA in young patients reported revision rates of 6.32% for HRA versus 6.14% for THA, a difference that was not statistically significant, alongside complication rates of 12.08% and 16.24%, respectively, with no between-group difference in functional outcomes [32]. The advantage of HRA at revision is rooted in the complete preservation of the femoral neck and proximal femur. However, the presumed reconstructive advantage is supported mainly by densitometric and biomechanical evidence, whereas direct comparative data from revision series remain limited. Densitometric data confirm a net BMD gain of approximately 34% across the proximal metaphysis at two years, providing a biologically optimized substrate for cementless stem osseointegration at conversion [3]. In published series, the femoral side of HRA revision is technically when the indication is an isolated femoral head or neck failure, permitting use of standard primary-length stems through the same surgical exposure. Nevertheless, the reconstructive value of HRA should not be assessed exclusively from the femoral perspective. While preservation of the femoral neck and metaphysis may simplify femoral reconstruction, long-term revision strategies also depend on acetabular bone stock, soft-tissue integrity and the cumulative impact of multiple future procedures [7].

### 3.2. Short-Stem Total Hip Arthroplasty

#### 3.2.1. Bone Preserving Rationale

Short-stem implants, conventionally defined by an overall length below 120 mm, represent a broadly applicable bone-preserving alternative to hip resurfacing arthroplasty, accommodating patients excluded from HRA candidacy by sex, head diameter or bone quality constraints. Their shared biomechanical denominator is metaphyseal load transfer through proximal fit-and-fill anchoring, leaving the femoral diaphysis entirely uninstrumented; the fixation principle and anatomical loading point, however, vary considerably across designs. Khanuja et al. proposed a classification into four types based on proximal loading location and fixation mechanism, demonstrating that surgical demands and tolerance for sizing errors increase progressively from shortened tapered designs (Type 4) toward neck-only fixation implants (Type 1) as showed in Table 2 [33].

This classification scheme is indispensable for interpreting cross-study comparisons, as the historical tendency to pool distinct designs has obscured clinically relevant differences in densitometric behavior and revision risk. The classification proposed by Falez further enriches this picture by distinguishing “collum”, “partial collum”, “trochanter-sparing” and “trochanter-harming” subtypes according to osteotomy level and fixation principle, each associated with distinct patterns of bone remodeling and complication profiles [34].

The JISRF (Joint Implant Surgery and Research Foundation) classification, proposed by McTighe et al., groups conservativve femoral implants by primary stabilization contact region rather than by stem length alone. Four zones are defined: head-stabilized designs, comprising hip resurfacing and mid-head resection; neck-stabilized designs, comprising short curved neck-sparing stems, lateral-flare engaging stems and neck-only implants; metaphyseal-stabilized designs, comprising tapered and bulky fit-and-fill stems; and conventional metaphyseal-diaphyseal stems. As displayed in Table 3, each zone maps to a specific anatomical contact area along the proximal femur, from the neck itself to the full diaphyseal canal and the scheme accommodates design variants, such as lateral-flare trochanteric engagement and isolated neck-plug fixation, that fall outside both the Khanuja and Falez systems [35].

Finite element analyses (FEA) have consistently demonstrated that metaphyseal-fixation short stems reduce proximal stress shielding compared with conventional diaphyseal designs. Yan et al. showed that, although both implant types, (Metha, B. Braun Aesculap) and (CLS, Zimmer-Biomet), decrease proximal femoral loading relative to the native femur, short stems generate significantly greater metaphyseal cortical stress and a more physiological load distribution than conventional stems (*p* < 0.0001), thereby attenuating the biomechanical conditions associated with proximal bone loss [36].

**Figure 1:**
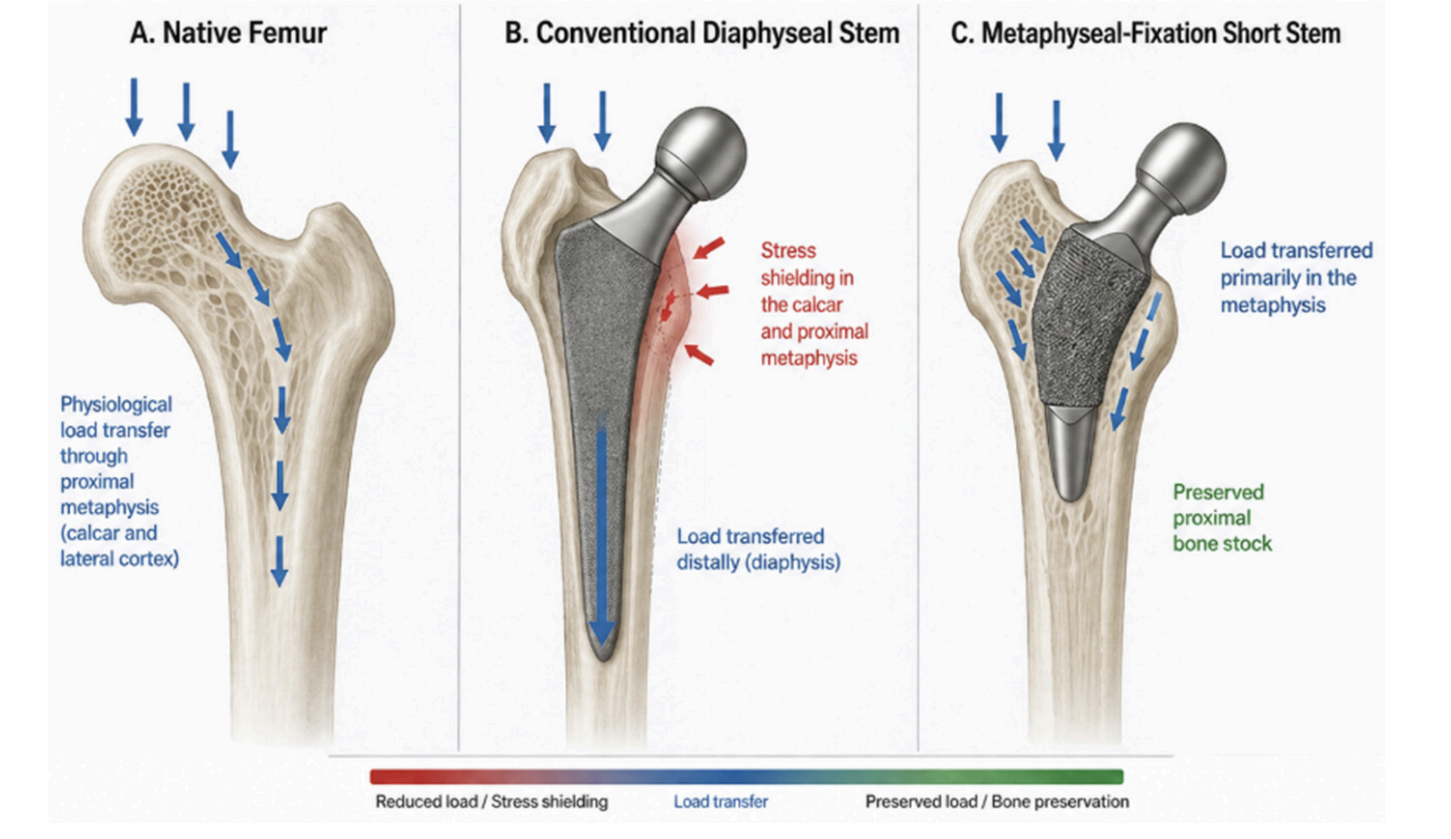
Biomechanical rationale of metaphyseal fixation in short-stem total hip arthroplasty. (**A**) Native femur showing physiological load transfer through the femoral head, calcar and lateral cortex, which maintains proximal bone homeostasis. (**B**) Conventional diaphyseal stem transfers a substantial proportion of the load distally, reducing mechanical stimulation of the proximal femur and leading to stress shielding, adaptive bone resorption and progressive loss of periprosthetic bone mineral density. (**C**) Metaphyseal-fixation short stem concentrates load transfer within the proximal femur, restoring a more physiological strain distribution, reducing stress shielding and promoting preservation of proximal femoral bone stock.

The biomechanical relevance of these findings lies in the mechanism of stress shielding. Under physiological conditions, the proximal femur is exposed to compressive and tensile loads that maintain bone homeostasis through adaptive remodeling. Conventional diaphyseal stems transfer load distally, reducing mechanical stimulation of the calcar and proximal femur and promoting stress shielding with subsequent bone resorption. In contrast, metaphyseal-fixation with short stems preserve a more physiological proximal load transfer, maintaining mechanical stimuli in the metaphysis and thereby limiting periprosthetic bone loss. FEA consistently correlates this more physiological strain distribution with the reduced proximal bone mineral density loss observed in long-term clinical studies, highlighting the importance of implant geometry and fixation strategy in preserving proximal femoral bone stock as shown in Figure 1 and Table 4 [33,34,36].

The clinical relevance of these findings is supported by long-term densitometric studies. At a mean follow-up of 13.3 years, a metaphyseal-anchoring short stem showed only 5.16% calcar bone loss in Gruen zone 7 compared with 33.04% observed with a conventional cobalt–chrome stem, while slight bone apposition was detected in zone 6, suggesting a more physiological pattern of load transfer [2]. Similarly, a randomized trial comparing an ultra-short titanium stem with a conventional tapered stem reported significantly lower proximal bone mineral density (BMD) loss in Gruen zone 1 at both 6 and 10 years, although clinical outcomes remained comparable between groups throughout follow-up [37].

Another remarkable stem is the Proxima (DePuy Synthes), which is a short lateral-engaging design with a sintered microbead surface for primary and secondary fixation. It corresponds to JISRF Class 2B and embodies the collum-type geometry that Burchard et al. identified as biomechanically closest to native femoral loading [35]. Finite element modelling comparing this stem with two other short-stem designs (Collo-MIS and Minima by LimaCorporate) found no significant difference in predicted remodeling pattern at two years, but showed that, under pronounced varus or valgus malpositioning, the Proxima stem alone developed localized cortical overload. This vulnerability follows from geometry rather than fixation principle: the short stem length concentrates strain at a single point of cortical contact once alignment departs from neutral, a pattern attributed to the point-contact profile inherent to this implant class [38].

The Minima stem (LimaCorporate), by contrast, occupies the metaphyseal-stabilized category (JISRF Class 3A) through a triple-tapered, squared cross-section engineered specifically for initial torsional stability and resistance to subsidence, combined with a porous titanium metaphyseal coating of 200 µm pore size for proximal osseointegration [39]. The stem itself is forged Ti6Al4V and fixation is not uniform along the implant: the proximal region carries the porous titanium coating for primary and secondary stability, while the distal surface is roughened separately via corundum-particle blasting. This distinction is missing in other types of stems and makes Minima a unique implant [40]. Digital image correlation and finite element validation against the shortened conventional Tri-Lock Bone Preservation Stem confirmed a proximal stress-shielding effect for both designs, but the strain distributions diverged: the Tri-Lock stem showed a greater lateral surface strain change relative to the non-implanted femur, an effect not reproduced by the Minima stem. The squared cross-section maintains wide cortical contact in Gruen zone 3 regardless of varus, neutral or valgus positioning, a property that plausibly explains the lower incidence of grade 1 or 2 stress shielding measured for this stem relative to the Tri-Lock design in a subsequent randomized comparison (28.9% versus 57.8%, *p* = 0.015). Unlike the Proxima stem, whose short length concentrates load at a single cortical point, the Minima geometry distributes strain across a broader contact area, a mechanism consistent with its tolerance for varus or valgus malalignment [41].

However, not all short-stem designs appear to behave similarly. In a prospective randomized study comparing the Nanos and Optimys stems, the Nanos implant showed significantly greater BMD loss in Gruen zones 1 (−10.1%, *p* = 0.001) and 7 (−21.3%, *p* = 0.001), whereas the Optimys stem demonstrated bone loss only in zone 7 (−16.2%, *p* = 0.001), together with a significant BMD increase in zone 5 (+6.8%, *p* = 0.001), highlighting the influence of implant design on periprosthetic remodeling [42].

Differences in biomechanical performance among short stems have also been confirmed by FEA studies. Burchard et al. demonstrated that, comparing seven different stem types (Silent, Metha, Nanos, Aida, Fitmore, SMF, Profemur Preserve) according to Falez classification plus a conventional reference stem (Spotorno standard), all short-stem designs produced less stress shielding than a conventional stem; however, only collum-type implants most closely reproduced native femoral biomechanics [43].

Beyond stem geometry, implant stiffness represents an additional determinant of load transfer. In a subsequent FEA study comparing three stem designs (Fitmore anatomical short, Ecofit Short straight, CLS standard) across three stiffness grades generated by hollow double-layer modelling, the author showed that reducing stem stiffness attenuates stress shielding across all designs, but that a statistically significant effect was achievable only with the anatomical short stem at its lowest stiffness level (Fitmore small shell versus full body: χ^2^ = 20.04, *p* < 0.001), supporting the preference for titanium alloys in bone-preserving arthroplasty. Owing to their lower elastic modulus (approximately 110 GPa versus 210 GPa for CoCr) compared with cobalt–chrome alloys, titanium stems could potentially promote more physiological load transmission and improved preservation of proximal femoral bone stock [44].

#### 3.2.2. Patient Selection

The optimal morphological substrate for short-stem THA is Dorr type A or B with intact proximal bone stock. Dorr type A, characterized by a narrow femoral canal, pronounced metaphyseal flare and thick cortices, is the canonical indication for calcar-guided designs: in this configuration, the proximal-to-distal mismatch encountered with conventional stems leads to distal cortical locking and calcar offloading that the short-stem geometry avoids by design. Although Dorr type C femoral morphology is generally considered a relative contraindication for calcar-guided implants because of reduced metaphyseal bone support, the available evidence is not uniform across all short-stem designs. In one implant-specific series, intraoperative periprosthetic fracture rates reached 22.2% in Dorr C femurs, compared with 2.1% and 0% in Dorr B and Dorr A femurs, respectively; however, these findings should not be generalized to all short-stem designs [33]. The target population spans both sexes aged 40–70 years with BMI below 35 kg/m^2^, though obesity alone does not preclude short-stem use when adequate metaphyseal fixation is confirmed intraoperatively [45]. Short-stem THA patients are on average younger than their conventional-stem counterparts, a pattern reflected in registry data, where the mean age of short-stem recipients was 63 versus 67 years for standard-stem patients [46].

Primary osteoarthritis constitutes the standard indication, yet the spectrum extends in experienced hands. Osteonecrosis with preserved metaphyseal bone quality is not a contraindication: cohort studies have confirmed reliable mid-term results and zero revisions for aseptic loosening in patients under 60 years, a favorable profile independently reproduced at minimum 10-year follow-up [47,48]. Mild acetabular dysplasia of Crowe grade I–II is addressable through resection-level adjustment in calcar-guided designs. Conversely, severe proximal femoral deformity, neck-shaft angles below 120°, anteversion exceeding 20° and post-traumatic metaphyseal compromise represent firm contraindications regardless of surgeon experience [33]. Not every short-stem design has proved equivalent safety across all indications: in a single-center series of 63 hips implanted with the Alteon Neck Preserving Stem (ANPS) followed for a mean of 46.6 months, the revision rate for femoral component loosening reached 6.3%, a figure the authors themselves characterized as unacceptably high, noting that radiographic subsidence was present in 9.7% of cases and that shelf formation complicated revision access in several instances. This experience reinforces the principle that implant selection within the short-stem category requires design-specific evidence and has to be tailored to the patient [49].

#### 3.2.3. Surgical Technique

Accurate preoperative digital templating is indispensable and must address neck resection level, target CCD angle, offset and limb length. Even when CCD angle reproduction is precise, systematic leg lengthening of approximately 5.00 mm (SD 5.98, *p* < 0.0001) relative to plan has been documented and must be anticipated prospectively [50]. The neck resection level is the primary driver of stem alignment: a high resection directs the implant into varus, preserving a larger femoral offset, while a low resection produces valgus positioning with correspondingly reduced offset. In valgus and neutral positions, the stem must be upsized until distal lateral cortical contact is secured, thus configuration is the most susceptible to undersizing and resulting subsidence. Lateral cortical contact, defined as a gap < 1 mm between stem tip and inner lateral cortex, represents the critical intraoperative endpoint: its absence correlates with significantly greater five-year axial migration (2.07 vs. 1.23 mm, *p* = 0.0018), independently of Dorr type or CCD category [51]. Incorrect sizing has been reported in 10.4–53.8% of cases across designs, with varus femurs (CCD categories A–B) carrying the highest risk due to the intraoperative tendency to pursue pronounced varus alignment before adequate cortical engagement is reached [52].

Femoral preparation follows the round-the-corner technique, in which curved rasps are introduced alongside the medial calcar in ascending sizes until cortical contact and stable fit-and-fill are achieved. With the direct anterior approach the greater trochanter is bypassed and gluteal muscles are almost completely spared. The osteotomy is performed in slight external rotation, with the lesser trochanter and fossa piriformis serving as landmarks for resection level. Intraoperative fluoroscopy in anteroposterior and axial planes may be useful after trial reduction to compare rasp positioning against the preoperative template and exclude undersizing before definitive implantation [53]. A systematic radiological control directly addresses the risk of coronal malalignment, which has been identified in neck-retaining designs in the early adoption phase and enables real-time verification of stem sizing, cortical contact and leg-length restoration [52,54].

The adoption of short-stem femoral components in total hip arthroplasty carries a reproducible learning curve regardless of surgical experience. Periprosthetic fracture rates of 2.7% have been reported, with 80% of events clustered within the first 30 cases, a finding consistent among residents in training [55,56]. Intraoperative adjustment rates decline markedly with experience, from 65.4% among residents to 32.7% in senior surgeons, with stem undersizing identified as the leading cause in 62% of cases [54]. On the other hand, varus stem positioning and leg length discrepancy persist beyond the phase of initial technical consolidation [57]. Of note, in the Alteon Neck Preserving Stem series, a 6.3% femoral revision rate was recorded despite a level 4/5 surgeon expertise rating on the Tang–Giddens scale, with no clustering of failures in the early operative experience—pointing to design-inherent technical demands beyond the conventional learning curve [49].

#### 3.2.4. Implant Survivorship

Evidence supporting the long-term reliability of short-stem total hip arthroplasty derives from systematic reviews, national arthroplasty registries and implant-specific cohort studies. At the highest level of evidence, a systematic review including 28 studies and 5322 hips showed a mean intraoperative fracture rate of 0.9%, neutral coronal alignment in 90.9% of cases and a combined implant survivorship of 98.6% at a mean follow-up of 12.1 years [58]. Registry data further corroborate these findings. Analysis of 232,269 THAs from the Dutch Arthroplasty Register demonstrated comparable 10-year revision rates between short and standard stems (4.8% versus 4.5%), with contemporary designs such as the Fitmore and Optimys performing similarly to conventional stems, whereas older-generation short stems showed significantly higher revision rates [46]. Likewise, data from the German Arthroplasty Register revealed lower revision rates for short stems between one and eight years postoperatively, while the Swiss registry documented a reduced expected failure rate up to 11 years after implantation [59]. When stratified by design category, partial-collum and trochanter-sparing stems consistently fulfilled the NICE benchmark of fewer than one revision per 100 observed component-years [34].

Long-term implant-specific studies have yielded similarly encouraging results. In a series of 72 hips implanted with the NANOS stem, survivorship reached 95.5% at a mean follow-up of 13.3 years, with only three revisions due to aseptic loosening, infection and periprosthetic fracture [48]. A second cohort of 118 NANOS stems proved survivorship rates of 98.3% and 97.5% at five and ten years, respectively, confirming the durability of neck-preserving designs and outcomes comparable to those reported for contemporary conventional stems.

Long-term data are similarly available for the Proxima stem: an all-cause revision rate of 2.4% (2 of 84 hips) corresponded to a 7-year Kaplan–Meier survival of 97.6%, while an independent 130-hip cohort reported 98.5% radiological and 100% clinical survival at a mean of nearly 10 years [60,61]. For the Minima stem, survivorship reached 100% at 3 years in 95 patients, when combined with a threaded Delta ST-C cup, and 100% clinical and radiological survival at a mean of 57 months in 18 hips with narrow femoral canals [39,40].

#### 3.2.5. Patient-Reported Outcomes

PROMs following short-stem THA are generally comparable to those seen after conventional THA. A meta-analysis of 12 randomized controlled trials including 1130 patients found no significant differences in Harris Hip Score (MD −0.38, 95% CI −1.02 to 0.26), revision rate (RR 1.52, 95% CI 0.71–3.26), or stem migration at one and two years [62]. Similarly, Axenhus et al. identified no significant differences in HHS or EQ-5D between ultra-short and conventional stems at either 6 or 10 years, despite superior proximal bone preservation in the short-stem group [37].

Long-term studies have also demonstrated sustained functional improvement following short-stem implantation. In a 25-year institutional experience including more than 943 implants, Harris Hip Score improved from 51.76 preoperatively to 92.86 at final follow-up, while monolithic metaphyseal stems showed a revision rate for aseptic loosening of only 0.78% at a mean follow-up of 5.2 years [59]. Comparable findings were observed for the NANOS stem, with HOOS improving from 32.25 ± 14.07 preoperatively to 91.91 ± 9.13 at a minimum 10-year follow-up, together with important gains in quality of life and sports-related function [48].

When compared with hip resurfacing arthroplasty (HRA), neck-preserving short-stem arthroplasty established similar patient-reported outcomes. In patients younger than 55 years, both MiniHip and HRA resulted in significant improvements in all HOOS domains, with no significant differences between groups at final follow-up [63]. Consistently, Riché et al. documented no significant differences in clinical outcomes among short stems, HRA and conventional stems [4].

Harris Hip Score improved from 40 to 91 points at 7 years with the Proxima stem and reached 98.8 points at final evaluation in an independent 130-hip cohort [60,61]. With the Minima stem, Harris Hip Score improved from a median of 46 to 96 points at 3 years and from 38.3 to 96.4 points at a mean of 57 months in patients with narrow femoral canals [39,40].

#### 3.2.6. Short-Stem-Specific Complications

Intraoperative periprosthetic fracture is the most common procedure-specific complication, with a mean rate of 0.9% across 5322 hips [58]; the calcar region is most vulnerable during impaction of oversized broaches and risk is disproportionately elevated in Dorr C femurs as described in Table 5. Among neck-preserving designs, the intraoperative fracture rate across 461 cases in the largest single-center long-term series reached 2.38%, yet none required subsequent revision when recognized and treated with cerclage intraoperatively [59]. These results are consistent, with the 3.3% rate of fracture reported for the CFP stem [52]. Component malalignment and undersizing remain interrelated technical complications: undersizing produces inadequate lateral cortex contact and is the primary modifiable contributor of early subsidence, while coronal malalignment across pooled series averaged 9.1% for varus deviation [33,63]. In specific anatomical scenarios like proximal femoral malunion, retained intramedullary hardware and prior osteotomy, short stems avoid corrective osteotomy entirely and reduce surgical morbidity without compromising fixation quality [64]. The performance profile of designs implanted through an anterior approach differs from those used via posterior access: short metaphyseal stems paired with the direct anterior approach facilitate femoral preparation by limiting the required neck resection level and simplifying access to the digital fossa, whereas neck-preserving implants can occasionally sustain partial anterior cortical damage during acetabular reaming [59]. Stress shielding in the proximal femur remains design-dependent. In a 10-year randomized controlled trial, the ultra-short stem group exhibited significantly less BMD reduction in Gruen zone 1 relative to conventional stems (mean difference −18.3%, 95% CI −28.0 to −9.0; *p* < 0.001), though zone 7 and overall periprosthetic BMD did not differ significantly between groups [37]. These findings align with systematic DEXA evidence showing approximately 5% bone loss in Gruen zone 1 and 10% in zone 7 at one year for short stems as a group, versus 8% and 14% for anatomic standard stems over the same interval [65]. Complications historically associated with modular neck designs, including titanium neck fatigue fracture and adverse local tissue reactions (ALVAL/pseudotumour) related to cobalt–chromium modular adapters, have become less common with contemporary monoblock metaphyseal stems, although outcomes remain partially design-dependent [59]. An outlier survivorship profile was observed with the Alteon Neck Preserving Stem, where femoral component survival was only 93.7% at a mean of 41.4 months in a single-center cohort of 63 hips, with 6.3% requiring revision for femoral loosening. The authors attributed this in part to the steep learning curve inherent to neck-only fixation designs and to distal shelf formation that complicated canal identification at revision [49].

Complication-specific data are available also for Proxima and Minima implants. In 86 Proxima hips, periprosthetic fracture occurred in 3.5% and malalignment in 12%, neither affecting clinical outcomes; an independent 130 hip series reported periprosthetic fracture in two hips and aseptic loosening with osteolysis in two hips (1.5%), both associated with moderate thigh pain [60,61]. In 18 Minima hips implanted in narrow femoral canals, intraoperative acetabular instability and canal underpreparation each occurred once, with heterotopic ossification in 11.1% of hips and no revisions at a mean of 57 months [39].

#### 3.2.7. Revision Rate and Revision Surgery

The Dutch Arthroplasty Register documents that femoral stem revisions attributed to periprosthetic fractures occur less frequently after short-stem THA (13%) than after standard-stem THA (24%), a finding consistent with the metaphyseal bone-preservation hypothesis [46]. At revision, short-stem THA may offer reconstructive advantages because preservation of the proximal femoral bone stock often allows implantation of a standard primary-length stem without the need for extensive femoral reconstruction or revision-specific components. Although this rationale is biologically plausible, it is supported predominantly by observational revision series and indirect evidence from bone-remodeling studies rather than by comparative clinical studies specifically evaluating revision complexity.

Consequently, bone defects are frequently limited to Paprosky type I or II lesions [66].

To facilitate the assessment of femoral bone loss in this setting, the Proximal Femoral Deficiency (PFD) classification has been proposed specifically for revisions of short-stem arthroplasty. The system stratifies defects according to the integrity of the femoral neck, metaphyseal cancellous bone and diaphysis, thereby supporting preoperative planning and implant selection. In its initial validation cohort of 21 cases, inter-observer agreement was considerable (κ = 0.71) and survivorship free from mechanical failure was 90.47% at a mean follow-up of 54 months (range 6–152 months). Although these early results are encouraging, further validation in larger cohorts is required before widespread adoption of the classification can be recommended [59,66].

## 4. Acetabular Bone-Sparing

### 4.1. Bone Preserving Rationale

Periacetabular bone loss in the context of contemporary cementless THA arises through two mechanistically distinct pathways, which are retroacetabular stress shielding and osteolysis secondary to particulate wear debris.

Contemporary modular press-fit cups are fabricated from titanium alloys with an elastic modulus of approximately 110 GPa, a value that contrasts sharply with native periacetabular cancellous bone (0.1–2 GPa). This mismatch concentrates load transfer at the equatorial rim, corresponding to DeLee and Charnley zones I and III, while selectively unloading the medial wall (zone II), thus causing retroacetabular stress shielding [5,67]. Clinical DXA studies have quantified the consequences: uncemented press-fit cups produce BMD decreases of 3% and 17% in the proximal and medial periacetabular regions, respectively over the first two postoperative years [68]. A combined finite element and strain gauge study further established that conventional titanium cups reduce cortical bone strains by 60–80% in the posterior-superior acetabular region, with bone loss accumulating to 90 mm^3^ across progressive stages of shielding and irremediable through surface coating modifications alone [69].

The second pathway, which is osteolysis secondary to particulate wear debris, operates independently of mechanical mismatch, driven by the biological response to bearing surface degradation products. Both mechanisms operate concurrently in vivo and their combined effect on periacetabular bone stock is the primary driver of acetabular reconstruction complexity at revision. The clinical significance of this observation is grounded in the Paprosky classification: as periacetabular defects progress from Type I to Type IIIB, technical requirements escalate from standard press-fit cups to rings and cages, cup-cage constructs and custom triflange components with porous metal augments or structural allografts, each step representing a disproportionate increase in surgical complexity and a corresponding reduction in long-term revision success [70].

### 4.2. Isoelastic Monoblock Cups and Porous Tantalum Components

Isoelastic monoblock acetabular components aim to reduce stress shielding by minimizing the mechanical mismatch between the implant and the surrounding bone. These components are manufactured from ultra-high molecular weight polyethylene (UHMWPE) or vitamin E-infused highly cross-linked polyethylene (VEHXLPE), materials characterized by an elastic modulus of approximately 0.5–1 GPa, which is closer to that of cancellous bone than conventional metallic shells. As a result, periacetabular load transfer is more physiological, promoting adaptive bone remodeling rather than stress shielding related resorption. To ensure biological fixation, the polyethylene shell is coated with a thin titanium layer that facilitates osseointegration while maintaining the overall low stiffness of the construct. The bone-preserving effect was quantified directly in a prospective RCT comparing the RM Pressfit vitamys isoelastic cup with a conventional modular titanium shell at four years using DXA: bone loss in the polar acetabular region (zone 2) was 4.9% ± 10.0% in the isoelastic group versus 15.9% ± 14.9% in the modular group (*p* = 0.005). This reduction in zone 2 bone loss translates into a lower Paprosky classification at future revision, with correspondingly simpler surgical options [71].

Porous tantalum monoblock acetabular components represent a distinct bone-preserving strategy based primarily on enhanced biological fixation rather than reduction of implant stiffness. Their highly porous trabecular structure, characterized by a porosity of approximately 75–80% and an average pore diameter of 550 μm, closely resembles cancellous bone architecture and provides a coefficient of friction higher than that of conventional porous-coated materials. These characteristics facilitate primary stability with lower impaction forces and promote rapid osseointegration through extensive bone ingrowth. In addition, the monoblock design eliminates backside polyethylene wear and avoids screw holes that could serve as pathways for particle migration and subsequent osteolysis. Consequently, porous tantalum components may contribute to long-term preservation of periacetabular bone stock through durable biological fixation rather than through an isoelastic load-transfer mechanism. In a prospective series of 151 primary THAs assessed at 8–10 years by serial EBRA-based radiographic analysis, complete absence of polyethylene wear, progressive radiolucencies, osteolytic lesions and component subsidence was documented, with radiological evidence of increased bone density and trabecular thickening in the periacetabular region at final follow-up [72].

### 4.3. Bearing Surface Tribology

Addressing the osteolytic pathway requires tribological optimization of the bearing surface. Ceramic-on-ceramic articulations, in particular fourth generation zirconia alumina composites, represent the most extensively validated strategy for debris elimination, combining superior hardness and wettability with a reduced fracture risk relative to earlier ceramic generations. Although ceramic bearings are not inherently bone-preserving from a structural or biomechanical perspective, the reduction in wear-related osteolysis indirectly supports long-term preservation of periprosthetic bone stock. In a retrospective series of 273 ceramic-on-ceramic THAs using BIOLOX delta bearings, at a mean follow-up of 12 years, showed no radiographic evidence of acetabular component loosening, liner fracture or osteolysis, with a bearing surface-related survivorship of 99.4% [73]. These outcomes were reproduced in a cohort of revision THAs with Delta ceramic-on-ceramic bearings, where no hip presented measurable wear or osteolysis and the mean modified Harris Hip Score improved from 53.3 preoperatively to 82.3 points at final follow-up, demonstrating the tribological durability of this bearing surface even in the mechanically demanding revision context [74]. Interesting results are shown also in ceramic-on-ceramic HRA. Cowie et al. demonstrated low wear under varied activities of daily function, with combined rates below 0.15 mm^3^/million cycles and no surface damage as highlighted in Table 6 [75]. Early clinical outcomes show 0.5% revision rates at two years, with no migration or osteolysis, and 45% return to high-impact sports [76].

VEHXLPE formulations provide an alternative or complementary tribological approach, particularly relevant in isoelastic cup systems, where the bearing material is integral to the cup structure. A prospective RCT showed a steady-state wear rate approximately 65% lower in the HXLPE/VitE group than in the UHMWPE group (*p* < 0.001) [77]. At the clinical level, a prospective study of 101 primary THAs using a cementless isoelastic monoblock cup with vitamin E-blended HXLPE reported a mean annual wear rate of 0.06 mm per year at five years, noticeably below the 0.1 mm/year threshold associated with osteolysis [78]. Taken together, the stress shielding attenuation achieved by isoelastic design and the osteolysis prevention obtained by optimized tribology operate through distinct and complementary pathways and their combination in a single implant configuration targets both mechanisms simultaneously.

### 4.4. Surgical Technique: Reaming Strategy and Component Positioning

Reaming diameter is the most directly modifiable intraoperative determinant of acetabular bone sacrifice and the relationship between reamer size and structural compromise is now biomechanically quantified. A pilot study on bone surrogates indicated that radial acetabular load-bearing capacity decreases from 3508 N at a reaming diameter of 54 mm to 2352 N at 58 mm, a statistically significant reduction attributable to progressive acetabular wall thinning [79]. This quantification supports the standard practice of under-reaming by 1–2 mm relative to component size, achieving adequate press-fit fixation while preserving the structural integrity of the acetabular rim. The single-reamer technique, in which one reamer prepares the acetabulum in a single anatomy-referenced step, further minimizes eccentric bone removal. In a retrospective analysis of 740 primary THAs, this approach achieved radiographic osseointegration in 99.7% at one year with a supplemental screw rate of only 3% and no intraoperative acetabular fractures [80].

Robotic-assisted acetabular preparation addresses the positioning accuracy dimension of bone conservation: cups placed outside the safe zone generate edge loading, accelerated wear and elevated debris generation that drive both osteolysis and cup migration. In a matched-pair controlled study comparing robotic-assisted with conventional posterior-approach THA, 100% of robotically placed cups fell within the safe zone defined by Lewinnek et al. while 80% of conventionally placed cups reach the same position. While 92% were within the more restrictive Callanan et al. modified safe zone compared with 62% in the conventional group. These data suggest that robotic guidance may reduce the long-term debris burden attributable to malpositioning, with downstream implications for periacetabular bone preservation [81,82].

### 4.5. Survivorship and Patient-Reported Outcomes

The clinical performance of isoelastic monoblock cup designs across independent series of varying size and follow-up length is consistent. The systematic review by Longobardi et al., encompassing 12 studies and 1491 hips at a mean follow-up of 8.1 years, confirmed overall survival of 82.7%, aseptic loosening-free survival of 94.4% and a mean Harris Hip Score of 92.6, with mean annual wear of 0.05 mm/year and VEHXLPE cups demonstrating significantly lower femoral head penetration than UHMWPE cups (*p* = 0.002) [5]. At 10-year follow-up in a prospective cohort of 162 hips implanted with the RM Pressfit vitamys, no acetabular lucent lines and no osteolysis were recorded on radiographic evaluation, with a mean modified Harris Hip Score of 94.8, a 10-year cumulative revision rate of 2.0% attributable exclusively to malpositioning and 100% survival with aseptic loosening as the endpoint [81]. In a series of 189 consecutive primary THAs with the RM Pressfit cup at mean follow-up of 6.5 years, no cup was revised for aseptic loosening and mean annual wear was 0.065 mm/year [82]. The prospective cohort study by Afghanyar et al. (n = 101, mean follow-up 5 years) using an isoelastic monoblock cup with vitamin E-blended HXLPE reported a mean annual wear rate of 0.06 mm/year, no osteolysis and no revision surgeries at mid-term follow-up [78].

### 4.6. Complications

The complication profile of isoelastic monoblock polyethylene cups is mechanistically shaped by the absence of dome holes, which precludes direct intraoperative confirmation of cup seating and exposes the polyethylene shell to deformation under excessive impaction force. In the largest available multicenter prospective cohort, 675 hips across nine institutions, 97.2% of cases were undertaken without intraoperative complication recorded; postoperative events over a follow-up extending to 8 years and 9 months included eight dislocations, three deep infections, 12 periprosthetic fractures and 10 nerve palsies. No acetabular osteolysis was observed in DeLee and Charnley zones 1–3 at any time point and a single nonprogressive radiolucent line was identified in zone 2 in one patient throughout the entire follow-up period [83]. These findings are consistent with the six-year RCT by Massier et al., in which 15 complications were recorded across 199 patients (8% per group), including six dislocations (3%), two perioperative femoral fractures in the vitamin E HXLPE arm and one postoperative acetabular fracture in the UHMWPE arm, all managed nonoperatively with full recovery; no adverse biological reactions attributable to the vitamin E additive were observed in either group [84]. Heterotopic ossification, though not a revision driver, was detected radiographically in up to 35 of 100 hips at ten-year follow-up in the Haefeli series and represented the most frequently recorded incidental finding across cohorts [81]. Osteolysis was documented in 3.2% of hips at ten years by Portet et al. and in eight hips in the long-term Ihle series, none of which had reached the clinical loosening threshold at final assessment [85,86].

### 4.7. Revision Rates and Revision Surgery

Survivorship across the isoelastic monoblock literature is determined by follow-up duration and polyethylene generation. In the Mahmood et al. multicenter cohort, Kaplan–Meier all-cause survival reached 98.9% at 105 months, with zero revisions for aseptic loosening; causes for the 13 recorded revisions were periprosthetic fracture (n = 3, including two stem-only procedures), prosthetic joint infection (n = 3), impingement (n = 1) and instability (n = 2) [83]. The RCT by Massier et al. confirmed an all-cause six-year survival of 98% for both the vitamin E HXLPE and UHMWPE monoblock cups, with 100% aseptic loosening-free survival in each arm [84]. At five to eight years, survival consistently exceeded 96% across independent cohorts: Erivan et al. documented 96.8% at a mean of 6.5 years and Massier et al. 100% aseptic loosening-free survival at a mean of 70 months follow-up with [82,84]. The single-surgeon comparative series by Kenanidis et al. reported six-year all-cause survival of 99.1% for the RM Pressfit vitamys versus 97.8% for the modular Pinnacle cup (log-rank *p* = 0.483), with equivalent aseptic loosening-free survival (log-rank *p* = 0.921), even in a monoblock cohort that was significantly younger (mean age 55.2 vs. 58.7 years, *p* = 0.002) [87]. The historical outlier remains the Ihle series, where 14 of 93 cups required revision at a mean of 12.3 years (15%), predominantly for osteolysis and aseptic loosening attributable to conventional UHMWPE wear debris; aseptic loosening-free survival in that cohort reached 94.4% at 19.8 years (95% CI 87–98), a failure mode not representative of contemporary cross-linked formulations [86].

For porous tantalum monoblock components, De Martino et al. showed 100% survivorship free of aseptic loosening at a minimum 15-year follow-up (mean 15.6 years) in 54 primary hips, with no radiographic evidence of loosening, migration, or gross polyethylene wear at final assessment [88]. These findings are corroborated by a recent large-cohort analysis, which proved excellent outcomes and survivorship with TMT monoblock cups in primary THA, reinforcing the long-term reliability of porous tantalum acetabular fixation. The Swedish registry analysis by Weiss et al. found no statistically significant difference in revision risk between 210 monoblock and 1130 modular cups (adjusted HR 2, 95% CI 0.8–6; *p* = 0.1), though the monoblock cohort was younger (median age 47 vs. 56 years, *p* < 0.001) and lacked access to highly cross-linked polyethylene, limiting direct inference [89].

From a surgical standpoint, the monoblock design carries specific implications when revision becomes necessary. The absence of a modular liner means that isolated liner exchange, a routine option with modular constructs, is not available, and full cup removal is required in the event of wear-related failure as briefly described also in Table 7. Conversely, the absence of osteolysis observed consistently across contemporary tantalum and isoelastic series suggests that, when revision is eventually undertaken, acetabular bone stock is likely to be well preserved [83,88]. This finding is of particular relevance in younger patients, for whom the quality of periacetabular bone at the time of a potential future revision is crucial. Sedel’s 30-year experience with ceramic-on-ceramic bearings further supports this principle. The near-absence of osteolysis in well-functioning alumina-on-alumina articulations was associated with preservation of periacetabular bone stock and reduced requirements for bone reconstruction at revision. However, revision of ceramic monoblock components remains technically demanding because complete cup removal is often necessary and may result in additional bone loss despite the absence of wear related osteolysis. [90].

## 5. Economic Burden of Bone-Sparing Techniques

Bone-sparing hip arthroplasty techniques demonstrate variable economic profiles depending on implant design and revision rates. Hip resurfacing arthroplasty (HRA), despite maximal bone preservation, has proven economically unfavorable compared to conventional total hip arthroplasty (THA). Economic modeling using National Joint Registry data revealed HRA incurred higher mean costs (incremental cost GBP 11,284) while generating fewer quality-adjusted life-years (−0.0879 QALYs), with THA demonstrating nearly 100% cost-effectiveness probability at any willingness-to-pay threshold [91]. Registry analysis confirmed only 60% of male and 2% of female HRA implants achieved the National Institute for Health and Care Excellence benchmark of 95% revision-free survival at 10 years, with overall HRA revision rates reaching 13% at 10 years compared to <4% for most THA devices [92].

Short-stem femoral prostheses present more favorable economics when appropriately selected. Registry data demonstrate comparable 12-year cumulative revision rates between short-stem (4.7%) and standard-stem (5.1%) THA, with short stems more frequently revised using standard-length rather than long stems (58% versus 46%), suggesting preserved bone stock facilitates less complex revisions [93]. However, survivorship varies by design, with certain short-stem models showing 10-year revision rates exceeding 6% compared to 4.5% for standard stems [46,94]. Neck-preserving stems demonstrate similarly heterogeneous outcomes, with several designs achieving excellent survivorship while others show prohibitive early failure rates that negate economic benefits [95].

Monoblock acetabular cups eliminate modularity-related complications but demonstrate comparable survivorship to modular designs. Swedish registry data showed 95% 5-year survivorship for monoblock cups versus 97% for modular designs, while a recent series reported 98.8% 10-year survivorship for any cup revision [89,96]. Systematic review found no difference in polyethylene wear rates, cup migration, or aseptic loosening between monoblock and modular cups, suggesting other factors should drive implant selection [97]. The economic impact of bone-sparing techniques must account for revision burden. Revision THA procedures cost USD 45,165–87,000 per episode compared to USD 25,615 for primary THA, with septic revisions costing 58% more than aseptic revisions [98]. First revision procedures carry approximately 20% probability of requiring second revision, creating a cascade effect that amplifies costs [99]. Consequently, any bone-sparing technique with elevated revision rates imposes long-term economic burden despite theoretical advantages for future revision surgery. Healthcare systems must weigh documented higher costs and revision risks against potential future benefits, with economic viability depending critically on careful patient selection and implant choice based on registry-validated survivorship data.

Although HRA provides the greatest degree of femoral bone preservation, contemporary arthroplasty strategies increasingly extend beyond the concept of bone preservation alone. Modern short-stem THA, particularly when combined with tissue-sparing surgical approaches such as the direct anterior approach, may preserve not only proximal femoral bone stock but also important soft-tissue structures involved in hip function and recovery. Consequently, the contemporary debate may no longer be limited to identifying the procedure that preserves the greatest amount of femoral bone, but rather the strategy that best preserves the overall functional hip unit while maintaining long-term reliability and future reconstructive flexibility.

## 6. Conclusions

Hip resurfacing arthroplasty (HRA) and contemporary short-stem total hip arthroplasty (THA) represent different strategies for bone preservation in young and active patients. Both aim to mitigate the biological consequences of proximal femoral bone loss and to facilitate future revision surgery, yet they differ substantially in indications, technical requirements, complication profiles and applicability across patient populations. Available evidence suggests that HRA may provide the greatest degree of femoral bone preservation and remains an attractive option in carefully selected patients, particularly young active males with favorable anatomy treated in experienced centers. Its use, however, is inherently limited by strict selection criteria, implant-specific complications and the continued need for specialized surgical expertise and postoperative surveillance. Contemporary short-stem THA, in contrast, is applicable to a wider range of patients and anatomical configurations. This category encompasses several implant philosophies, including neck-preserving, calcar-guided and shortened tapered designs, each characterized by distinct fixation principles, biomechanical behavior and clinical indications. Although many contemporary designs have shown encouraging medium-term and long-term results, these findings should not be generalized to short stems as a whole, and therefore should be interpreted design by design.

On the acetabular side, advances in implant design and bearing technology have extended the concept of bone preservation beyond the femur alone. Isoelastic cup designs and modern low-wear bearing surfaces may contribute to acetabular bone preservation and to the maintenance of reconstructive options at the time of future revision, although the available series are predominantly of intermediate follow-up.

Preservation of bone stock should not be regarded as an isolated endpoint. The goal of arthroplasty in young and active patients extends beyond retaining the greatest amount of bone and should instead focus on preserving the functional and reconstructive potential of the entire hip joint. Implant selection should therefore be individualized, balancing bone preservation, soft-tissue preservation, long-term reliability, functional recovery and future revision flexibility according to patient characteristics and implant-specific evidence. Individualized implant selection and positioning may optimize metaphyseal fixation, reduce stress shielding and preserve proximal bone stock, whether long-term outcomes improvements with the use of advanced planning tools and robotic assistance remains to be established.

Future research should move beyond isolated measures of femoral bone preservation and adopt joint-level, functional and reconstructive endpoints that better reflect the lifetime needs of young and active patients undergoing hip arthroplasty.

Given the narrative design of this review and the absence of a formal quality or risk-of-bias assessment, these conclusions should be interpreted as a structured synthesis of the available literature rather than as definitive evidence supporting the superiority of one bone-preserving strategy over another. Further high-quality comparative studies are required to clarify the long-term role of specific implant designs.

## Figures and Tables

**Table 1:** Patient selection criteria for femoral bone-sparing hip arthroplasty.

Criterion	Short-Stem Total Hip Arthroplasty	Hip Resurfacing Arthroplasty (HRA)
**Ideal candidate**		
**Sex**	Both sexes	Male preferred; female: relative CI (ARMD risk, smaller head diameter)
**Age**	40–70 years	<65 years; optimal <55 years
**Diagnosis**	Primary OA; AVN with intact metaphysis; mild dysplasia (Crowe I–II)	Primary OA (first choice); AVN < 1/3 femoral head
**Femoral head diameter**	-	≥48 mm mandatory; ≥55 mm optimal; <44 mm: >5× revision risk
**Femoral morphology**	Dorr type A (ideal); B (acceptable); Dorr C: high fracture risk	Head and neck bone quality; cysts > 1 cm: absolute CI
**Bone mineral density**	Intact metaphyseal bone stock mandatory	Normal BMD required (T-score > −2.5)
**Activity level**	Moderate to high activity	High-impact athletes; RTS rate 90.9% at 4 years
**Centre volume**	Learning curve ~30–50 cases	>100 cases/year; personal learning curve ≥ 100 cases
**Absolute contraindications**		
-	Dorr C; CCD angle < 120°; femoral anteversion > 20°; post-traumatic metaphyseal compromise; severe proximal deformity	Renal insufficiency; metal hypersensitivity; T-score < −2.5; AVN > 1/3 femoral head; femoral cysts > 1 cm; DDH/SCFE/Perthes

CI: contraindication; OA: osteoarthritis; AVN: avascular necrosis; DDH: developmental dysplasia of the hip; SCFE: slipped capital femoral epiphysis; BMD: bone mineral density; ARMD: adverse reaction to metal debris; RTS: return to sport; CCD: centrum-collum-diaphysis angle.

**Table 2:** Khanuja classification.

Type	Subtypes	Description
**Type 1**	1A Wedge-shaped; 1B Cylindrical; 1C Threaded	Femoral neck-only fixation
**Type 2**	2A Trapezoidal; 2B Rounded; 2C Threaded; 2D Thrust plate	Calcar-loading fixation
**Type 3**	Lateral-flare designs	Calcar-loading with lateral flare
**Type 4**	Shortened tapered stems	Shortened tapered conventional stems

**Table 3:** JISRF (Joint Implant Surgery and Research Foundation) classification.

Type	Subtype
**Type 1—Head-stabilized fixation**	1A Hip resurfacing
	1B Mid-head resurfacing
**Type 2—Neck-stabilized fixation**	2A Short curved neck-sparing
	2B Short lateral-flare engaging
	2C Neck plugs/neck-only
**Type 3—Metaphyseal-stabilized fixation**	3A Tapered
	3B Bulky/fit-and-fill
**Type 4—Conventional metaphyseal-diaphyseal fixation**	—

**Table 4:** Bone-preservation advantages of femoral bone-sparing techniques.

Parameter	Short-Stem THA	HRA
**Bone preservation**		
**Mechanism**	Metaphyseal fixation; diaphysis uninstrumented	Retains femoral head and neck entirely; no canal preparation
**Periprosthetic BMD**	Calcar atrophy 5.16% (zone 7) vs. 33.04% diaphyseal CoCr at 13 years	+34% BMD gain at proximal metaphysis at 2 years
**Stress shielding vs. conventional stem**	Zone 1: −18.3% vs. −36% conventional (*p* < 0.001) at 10 years	None (net densitometric gain)
**Survivorship**		
**10-year implant survival**	98.6% pooled (systematic review; n = 5322; mean follow-up 12.1 years)	95% (males ≤ 50 years; n = 11,382; 27 centers)
**15-year implant survival**	NANOS: 95.5% at mean 13.3 years; Metha: 95.50%	95.1% in men; 95.8% for head ≥ 48 mm; 88.98% across all comers
**22-year implant survival**	-	90% (well-performing designs; low-risk males)
**Functional outcomes**		
**Harris Hip Score**	MD −0.38 vs. conventional (95% CI −1.02 to 0.26; *p* = 0.89)	93.9 (median) at 12.4 years
**Return to sport**	Equivalent to HRA; superior to conventional THA	90.9% at 4 years; Ironman athletes 94%
**Dislocation rate vs conventional THA**	Comparable to conventional THA	RR 13.45× lower (95% CI 3.37–98.21)
**Revision implications**		
**Bone stock at revision**	Paprosky I–II in most cases; standard stem without ETO	Optimal: net BMD gain; standard primary stem at conversion without ETO

HRA: hip resurfacing arthroplasty; BMD: bone mineral density; CoCr: cobalt–chromium; ETO: extended trochanteric osteotomy; MD: mean difference; NANOS: Neck Arthroplasty by Natural Osteotomy Support.

**Table 5:** Procedure-specific complications of femoral bone-sparing techniques.

Complication	Short-Stem THA	HRA
	***Class-specific***	
**ALVAL/ARMD/metallosis**	Not applicable (monoblock titanium designs)	With MoM bearing: pseudotumour; soft-tissue necrosis; Co elevated 82%, Cr 53% of failed MoM
**Femoral neck fracture**	-	0.5–2.0%; peak within 6 months; notching reduces retinacular flow ~50%
**Intraoperative periprosthetic fracture**	0.9–2.4% overall; Dorr C rate 22.2%; 80% within first 30 cases	Rare; neck notching primary risk
**Component malalignment/undersizing**	Varus deviation 9.1% pooled; undersizing 10.4–53.8% across designs	Varus positioning: primary modifiable risk for neck fracture
**Early subsidence**	Axial migration 2.07 vs. 1.23 mm (*p* = 0.0018) without lateral cortex contact	-
**Metal ion elevation**	None with monoblock titanium designs	Systemic Co/Cr elevation; multimodal surveillance required—serum ions + MARS-MRI
	***Residual stress shielding***	
**Zone 1 (lateral metaphysis)**	−18.3% vs. −36% conventional at 10 years; ~5% at 1 year vs. ~8% standard stems	None: net BMD gain
**Zone 7 (calcar)**	~10% at 1 year (design-dependent)	None: net BMD gain
	***Learning curve***	
**Component positioning errors**	Fractures 80% within first 30 cases; adjustment rate 65.4% residents vs. 32.7% seniors	Elevated in centers < 100 cases/year; CAS reduces outliers 48% → 33%
**Outlier design risk**	ANPS stem: 6.3% revision for loosening at mean 41.4 months; subsidence 9.7%	Poorly performing MoM designs: significantly higher early revision rates

HRA: hip resurfacing arthroplasty; ALVAL: aseptic lymphocyte-dominated vasculitis-associated lesion; ARMD: adverse reaction to metal debris; MoM: metal-on-metal; Co: cobalt; Cr: chromium; MARS-MRI: metal artefact reduction sequence MRI; BMD: bone mineral density; CAS: computer-assisted surgery; ANPS: Alteon Neck Preserving Stem.

**Table 6:** Bone-preservation advantages of acetabular bone-sparing techniques.

Parameter	Porous Tantalum Monoblock Cup	Isoelastic Monoblock Cup (VEHXLPE/UHMWPE)
	**Bone preservation mechanism**	
**Design principle**	Porosity 75–80%; pore diameter 550 µm; coefficient of friction ≈ 2 × conventional sintered porous coatings	Elastic modulus ≈ cancellous bone (0.5–1 GPa); reduces stress shielding at source
**Periacetabular BMD (zone 2)**	Progressive BMD increase and trabecular thickening at 8–10 years	Loss 4.9% ± 10.0% vs. 15.9% ± 14.9% modular (*p* = 0.005) at 4 years
**Osteolysis prevention**	Zero osteolysis in all available series at 8–15 years	VEHXLPE: wear 65% lower than UHMWPE (*p* < 0.001); annual wear 0.05–0.06 mm/year
**Backside wear**	Absent (monoblock; no modular interface)	Absent (monoblock; no modular interface)
	**Survivorship**	
**All-cause survival**	100% aseptic loosening-free at minimum 15 years (mean 15.6 years; n = 54)	98.9% at 105 months (n = 675, 9 centers); 98% at 6 years in RCT (n = 199)
**Aseptic loosening-free survival**	100% at minimum 15 years	100% in contemporary VEHXLPE series at 6–10 years
	**Functional outcomes**	
**mHHS**	-	92.6 (systematic review of 12 studies; n = 1491)
**Annual wear rate**	No measurable gross PE wear at 15 years	0.05–0.06 mm/year; below 0.1 mm/year osteolysis threshold
	**Revision implications**	
**Bone stock at revision**	Well preserved: near-absent osteolysis at retrieval in all available series	Well preserved: near-absent osteolysis; lower Paprosky grade vs. modular cups

VEHXLPE: vitamin E-infused highly cross-linked polyethylene; UHMWPE: ultra-high molecular weight polyethylene; BMD: bone mineral density; GPa: gigapascal; PE: polyethylene; mHHS: modified Harris Hip Score; RCT: randomized controlled trial.

**Table 7:** Procedure-specific complications of acetabular bone-sparing techniques.

Complication	Porous Tantalum Monoblock Cup	Isoelastic Monoblock Cup
	**Intraoperative**	
**Cup seating verification**	Standard impaction; resistance confirms primary stability; 97.2% cases without intraoperative complication (n = 675)	Not directly confirmable (no dome holes); deformation risk under excessive impaction
**Acetabular fracture**	-	1 postoperative case in RCT (n = 199)
	**Postoperative**	
**Dislocation**	Not specifically elevated	8/675 hips (1.2%) at 8.75 years
**Deep infection**	<2% across all series	<2% across all series (consistent with registry data)
**Osteolysis**	Zero in all available series at 8–15 years	3.2% at 10 years (conventional UHMWPE) absent with VEHXLPE/CoC in contemporary series
**Heterotopic ossification**	-	Up to 35% radiographically at 10 years (not a revision driver)
**Residual stress shielding**	No measurable stress shielding (progressive BMD increase)	Significantly reduced vs modular cups across all DeLee–Charnley zones
	**Revision-specific**	
**Liner exchange**	Monoblock design: isolated liner exchange not available; full cup removal required at revision	Monoblock design: isolated liner exchange not available; full cup removal required at revision
**Historical UHMWPE failure**	Not applicable	15% revision at mean 12.3 years (osteolysis/loosening with conventional UHMWPE); not applicable with VEHXLPE

UHMWPE: ultra-high molecular weight polyethylene; VEHXLPE: vitamin E-infused highly cross-linked polyethylene; CoC: ceramic-on-ceramic; BMD: bone mineral density; RCT: randomized controlled trial.

## Data Availability

No new data were created or analyzed in this study.
